# A publicly available benchmark for biomedical dataset retrieval: the reference standard for the 2016 bioCADDIE dataset retrieval challenge

**DOI:** 10.1093/database/bax061

**Published:** 2017-08-18

**Authors:** Trevor Cohen, Kirk Roberts, Anupama E. Gururaj, Xiaoling Chen, Saeid Pournejati, George Alter, William R. Hersh, Dina Demner-Fushman, Lucila Ohno-Machado, Hua Xu

**Affiliations:** 1 School of Biomedical Informatics. The University of Texas Health Science Center at Houston/7000 Fannin St. Suite 600, Houston, TX, 77030, USA; 2 Population Studies Center, University of Michigan, 426 Thompson St. Ann Arbor, MI, 48104, USA; 3 Department of Medical Informatics and Clinical Epidemiology, Oregon Health and Science University, 3181 S.W. Sam Jackson Park Rd, Portland, OR, 97239, USA; 4 U.S. National Library of Medicine, 8600 Rockville Pike, Bethesda, MD 20894 USA; 5 Department of Biomedical Informatics, University of California San Diego, Altman Clinical and Translational Research Institute Building, 9452 Medical Center Drive, La Jolla, CA, 92093, USA

## Abstract

The rapid proliferation of publicly available biomedical datasets has provided abundant resources that are potentially of value as a means to reproduce prior experiments, and to generate and explore novel hypotheses. However, there are a number of barriers to the re-use of such datasets, which are distributed across a broad array of dataset repositories, focusing on different data types and indexed using different terminologies. New methods are needed to enable biomedical researchers to locate datasets of interest within this rapidly expanding information ecosystem, and new resources are needed for the formal evaluation of these methods as they emerge. In this paper, we describe the design and generation of a benchmark for information retrieval of biomedical datasets, which was developed and used for the 2016 bioCADDIE Dataset Retrieval Challenge. In the tradition of the seminal Cranfield experiments, and as exemplified by the Text Retrieval Conference (TREC), this benchmark includes a corpus (biomedical datasets), a set of queries, and relevance judgments relating these queries to elements of the corpus. This paper describes the process through which each of these elements was derived, with a focus on those aspects that distinguish this benchmark from typical information retrieval reference sets. Specifically, we discuss the origin of our queries in the context of a larger collaborative effort, the biomedical and healthCAre Data Discovery Index Ecosystem (bioCADDIE) consortium, and the distinguishing features of biomedical dataset retrieval as a task. The resulting benchmark set has been made publicly available to advance research in the area of biomedical dataset retrieval.

**Database URL:** https://biocaddie.org/benchmark-data

## Introduction

Biomedical research is increasingly driven by the acquisition and analysis of large digital datasets (a dataset is an organized collection of data, usually concerning the results of a study or experiment). Sharing of such datasets is encouraged as a means to promote reproducibility (1), and to extract further value from existing biomedical data, which are generally far more expensive to collect than to analyse. However, the proliferation of publicly available data presents new challenges to the biomedical researcher, as datasets relevant to a problem of interest may be scattered across multiple repositories, and indexed inconsistently. This presents a barrier to sharing of data in general, and to sharing of data across research communities in particular. There is a pressing unmet need for the development of new methods to enable biomedical researchers to find relevant datasets within this rapidly expanding information ecosystem. To this end, the biomedical and healthCAre Data Discovery Index Ecosystem (bioCADDIE) consortium (2), funded by the NIH Big Data to Knowledge (BD2K) program (3), aims to enable researchers to find data from sources other than those they would usually encounter. A component of this project involves the development and evaluation of DataMed (4, 5), a publicly-available prototype search engine for biomedical datasets. At the time of this writing, DataMed has indexed 1 375 977 datasets drawn from 66 repositories (not all of these datasets are included in the titular reference standard, which was constructed in March of 2016), with plans to further broaden the range and quantity of data available underway. In order to establish the performance of existing information retrieval methods in this novel problem domain, and encourage the rapid development of new methods for this purpose, the bioCADDIE consortium orchestrated a shared task–the 2016 bioCADDIE Dataset Retrieval Challenge (6). In this paper, we describe the reference standard for this challenge, which we have made publicly available for the purpose of encouraging research in the nascent field of biomedical dataset retrieval.

## Background

The need for novel methods to enhance the indexing of biomedical data available in public repositories [a dataset repository is a collection of individual datasets, which in biology is often focused on a particular domain (e.g. “The Cancer Imaging Archive”) or organism (e.g. FlyBase)] has been recognized for some time, and efforts have been made to enhance specific indexes (a dataset index is a structure intended to enable search and retrieval of datasets, within or across repositories) using informatics methods for the purpose of integration across repositories (7–10). The main thrust of these efforts has been on the development of automated solutions to the problem of standardization of metadata elements. This is an important concern for retrieval and integration of biomedical datasets, as both the structured and unstructured components of the descriptive metadata harvested from source repositories vary considerably in terminology and granularity. However, the challenges presented by the task of biomedical dataset retrieval go beyond the normalization of metadata elements. The information required to determine whether or not a particular dataset meets a stated information need may not be present in these metadata fields at all, so review of associated literature, or the dataset itself, is required before an accurate assessment of utility can be made. Thus, there is the need for further research to establish the utility of existing information retrieval methods and promote the development of novel approaches to this problem. It is our intention that the release of this publicly available reference benchmark will facilitate these ends, as the sharing of similarly structured reference standards has advanced the science of information retrieval in both the general (11) and biomedical domains (12–17).

Although a detailed review of the history of challenge evaluations in biomedical retrieval is beyond the scope of the current paper [we refer the interested reader to Hersh (18) for a thorough account of the history of this field up to 2008, and to Voorhees (17) and Roberts *et al.* (14) for accounts of the more recent Medical Records and Clinical Decision Support tracks at TREC], we provide here a brief account of the reference sets of past challenges involving biomedical documents indexed with both structured and unstructured data, for the purpose of comparison with the current benchmark. The corpus for the TREC Medical Records track consisted of 93 551 de-identified medical reports, which were gathered from the University of Pittsburgh’s Natural Language Processing (NLP) repository. These reports include both structured components (coded diagnoses) and unstructured components (narrative text notes) (17). Similarly, as the TREC Genomics and TREC CDS corpora were derived from PubMed and PubMed Central, respectively, (12, 14), they include both structured data (in the form of Medical Subject Heading terms) and narrative text. However, in all of these cases the corpus concerned was indexed with a single vocabulary, and the number of structured fields was small. As we shall describe, the components of our current corpus are drawn from across multiple repositories, and as such are terminologically inconsistent with respect to both field name and entity. Furthermore, the narrative text descriptions provided are often terse, and may concern the research findings of an associated publication, rather than the data set itself. These issues present additional challenges for information retrieval systems, and an important motivation for the bioCADDIE challenge was the need for new technologies to address them.

## Corpus

As of March 24th 2016, DataMed had harvested datasets from 20 public repositories following the procedure described in (4), resulting in a collection of 794 992 datasets (Table 1). The rationale for selection of these repositories is documented in the publicly available bioCADDIE white paper (19), and was based on the recommendations of a joint workshop involving both the Big Data to Knowledge (BD2K) consortium, and ELIXIR, a European Distributed Infrastructure for Life-Science Information. Priorities for identified for inclusion in the initial index included specific databases (e.g. Protein Databank) and those repositories utilized by the BD2K centers, and by a set of BD2K-funded administrative supplements. This collection serves as the corpus of the challenge. The corpus contains metadata fields from original repositories, with some additional cleansing including removal of redundant and invalid records–any empty datasets; datasets that did not have a repository associated with them (as these were test cases used in the development process); and outdated datasets (where a more recent version was also indexed). A unique dataset identifier was assigned to every document for evaluation purposes. Each dataset reference in the corpus includes a unique document identifier (DOCNO), title (TITLE), repository identifier (REPOSITORY) and a nested ‘metadata’ field (METADATA), containing structured and unstructured information harvested from the description of each dataset on its source repository. Frequently occurring metadata subfields include ‘gene’, ‘organism’, ‘keywords’ and ‘citation’. For some records, a PubMed identifier (PMID) is also available, which can be used to link the data to a related publication. The resulting metadata were stored in both XML and JSON formats. The corpus is publicly available at https://biocaddie.org/biocaddie-2016-dataset-retrieval-challenge.

**Table 1.** Repositories harvested to generate the corpus of dataset metadata

Arrayexpress (60 881)	ArrayExpress Archive of Functional Genomics Data stores data from high-throughput functional genomics experiments, and provides these data for reuse to the research community.
Bioproject (155 850)	A BioProject is a collection of biological data related to a single initiative, originating from a single organization or from a consortium. A BioProject record provides users a single place to find links to the diverse data types generated for that project.
The cancer imaging archive (63)	The Cancer Imaging Archive (TCIA) is a large archive of medical images of cancer accessible for public download. All images are stored in DICOM file format. The images are organized as ‘Collections’, typically patients related by a common disease (e.g. lung cancer), image modality (MRI, CT, etc) or research focus.
Clinicaltrials (192 500)	ClinicalTrials.gov is a registry and results database of publicly and privately supported clinical studies of human participants conducted around the world.
Clinical trials network (46)	A repository of data from completed CTN clinical trials to be distributed to investigators in order to promote new research, encourage further analyses, and disseminate information to the community. Secondary analyses produced from data sharing multiply the scientific contribution of the original research.
Cardiovascular research Grid (29)	The CardioVascular Research Grid (CVRG) project is creating an infrastructure for sharing cardiovascular data and data analysis tools. CVRG tools are developed using the Software as a Service model, allowing users to access tools through their browser, thus eliminating the need to install and maintain complex software.
Dataverse (60 303)	A Dataverse repository is the software installation, which then hosts multiple dataverses. Each dataverse contains datasets, and each dataset contains descriptive metadata and data files (including documentation and code that accompany the data). As an organizing method, dataverses may also contain other dataverses.
Dryad (67 455)	DataDryad.org is a curated general-purpose repository that makes the data underlying scientific publications discoverable, freely reusable, and citable.
Gemma (2285)	Gemma is a web site, database and a set of tools for the meta-analysis, re-use and sharing of genomics data, currently primarily targeted at the analysis of gene expression profiles. Gemma contains data from thousands of public studies, referencing thousands of published papers.
Gene expression omnibus (105 033)	Gene Expression Omnibus is a public functional genomics data repository supporting MIAME-compliant submissions of array- and sequence-based data. Tools are provided to help users query and download experiments and curated gene expression profiles.
Mouse phenome database (235)	The Mouse Phenome Database (MPD) has characterizations of hundreds of strains of laboratory mice to facilitate translational discoveries and to assist in selection of strains for experimental studies.
Neuromorpho (34 082)	NeuroMorpho.Org is a centrally curated inventory of digitally reconstructed neurons associated with peer-reviewed publications. It contains contributions from over 80 laboratories worldwide and is continuously updated as new morphological reconstructions are collected, published, and shared.
Nuclear receptor signaling atlas (NURSA) (389)	The Nuclear Receptor Signaling Atlas (NURSA) was created to foster the development of a comprehensive understanding of the structure, function, and role in disease of nuclear receptors (NRs) and coregulators. NURSA seeks to elucidate the roles played by NRs and coregulators in metabolism and the development of metabolic disorders (including type 2 diabetes, obesity, osteoporosis, and lipid dysregulation), as well as in cardiovascular disease, oncology, regenerative medicine and the effects of environmental agents on their actions.
Openfmri (36)	OpenfMRI.org is a project dedicated to the free and open sharing of functional magnetic resonance imaging (fMRI) datasets, including raw data. The focus of the database is on task fMRI data.
Peptideatlas (76)	PeptideAtlas is a multi-organism, publicly accessible compendium of peptides identified in a large set of tandem mass spectrometry proteomics experiments. Mass spectrometer output files are collected for human, mouse, yeast, and several other organisms, and searched using the latest search engines and protein sequences.
Phenodisco (dbGaP) (429)	Phendisco is derived from the database of Genotypes and Phenotypes (dbGap), with additional metadata (9).
Physiobank (70)	PhysioBank is a large and growing archive of well-characterized digital recordings of physiologic signals and related data for use by the biomedical research community. PhysioBank currently includes databases of multi-parameter cardiopulmonary, neural, and other biomedical signals from healthy subjects and patients with a variety of conditions with major public health implications, including sudden cardiac death, congestive heart failure, epilepsy, gait disorders, sleep apnea, and aging.
Protein data bank (113 493)	The Protein Data Bank (PDB) archive is the single worldwide repository of information about the 3D structures of large biological molecules, including proteins and nucleic acids found in all organisms including bacteria, yeast, plants, flies, other animals, and humans.
ProteomeXchange (1716)	The ProteomeXchange consortium has been set up to provide a single point of submission of MS proteomics data to the main existing proteomics repositories, and to encourage the data exchange between them for optimal data dissemination.
Yale protein expression database (21)	The Yale Protein Expression Database (YPED) is an open source system for storage, retrieval, and integrated analysis of large amounts of data from high throughput proteomic technologies. YPED currently handles LCMS, MudPIT, ICAT, iTRAQ, SILAC, 2D Gel and DIGE, Label Free Quantitation (Progenesis), Label Free Quantitation (Skyline), MRM analysis and SWATH This repository contains data sets which have been released for public viewing and downloading by the responsible Primary Investigators.
Total (794 992)	

The numbers in parentheses indicate the number of datasets in each repository when the corpus was constructed.

A sample document is provided in XML format in Table 2 below. A number of the subfields are represented in the <METADATA> field, though the nature and distribution of these subfields varies considerably across the corpus.

**Table 2.** Sample Dataset in XML format

**<DOC>** **<DOCNO>**6408**</DOCNO>** **<TITLE>**Vitamin D receptor (VDR) target genes in THP-1 monocytic leucemia cells**</TITLE>** **<REPOSITORY>**arrayexpress_020916**</REPOSITORY>** **<METADATA>**{"dataResource": {"*keywords*": [], "*altNames*": [], "*acronyms*": []}, "*citation*": {"*count*": "0"}, "*organism*": {"*experimen*t": {"*species*": "Homo sapiens"}}, "dataItem": {"*description*": "The biologically active form of vitamin D, 1,25-dihydroxyvitamin D3 (1,25(OH)2D3), is a direct regulator of gene transcription, since it is the only high affinity natural ligand of the transcription factor vitamin D receptor (VDR). Transcriptome-wide analysis of THP-1 human monocyte-like cells had indicated more than 600 genes to be significantly (p < 0.05) regulated after a 4 h stimulation with 1,25(OH)2D3. In this study, we screened of the list of primary vitamin D targets for genes encoding for transcriptional regulators and selected those of the activating transcription factor NFE2 and the transcriptional repressor BCL6. Both genes are under the control of two VDR loci and are the only 1,25(OH)2D3 targets within their respective chromosomal domain. However, NFE2 mRNA was rapidly up-regulated, while the increase of BCL6 expression showed a slower rise. After 24 h incubation of THP-1 cells with 1,25(OH)2D3 more than 1,500 genes responded significantly (p < 0.001), of which 132 where more than 2-fold induced. Public chromatin immunoprecipitation-sequencing datasets suggested that the majority of these genes could be targets of NFE2 or BCL6. In time course experiments we displayed for representative gene examples the specific delayed response of secondary 1,25(OH)2D3 targets and confirmed for the respective chromosomal domains the genomic binding of NFE2, BCL6 and VDR. In conclusion, our study indicated that the physiological response of monocytes to 1,25(OH)2D3 involves the action of NFE2 and BCL6. THP-1 cells were treated 24 h either with 0.1% ethanol (vehicle, control) or 1\u03b1,25(OH)2D3 (1,25D)", "title": "Vitamin D receptor (VDR) target genes in THP-1 monocytic leucemia cells", "*releaseDate*": "2015-04-26", "*lastUpdateDate*": "2015-05-02", "*dataTypes*": ["*organism*", "*dataItem*", "*citation*"], "ID": "522721", "*experimentType*": "transcription profiling by array"}}**</METADATA > </DOC>**

Main fields are in boldface, and subfields are in *italics.*

## Query creation

**Figure 1.**
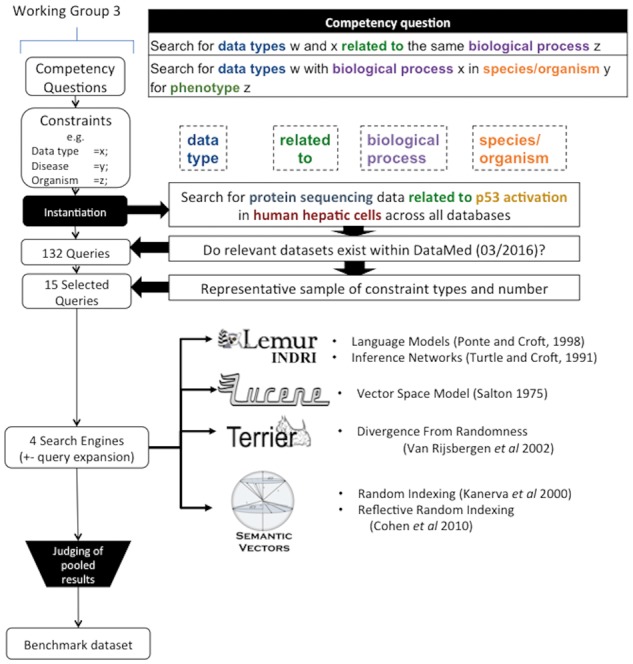
Overview of construction of the reference standard.

The remaining components of the reference standard consist of the queries, and the relevance judgments for datasets represented in the corpus in relation to these queries. Figure 1 provides an overview of the process through which these components were created. The queries were developed from a set of competency questions that in turn were based on use cases proposed by stakeholders affiliated with the bioCADDIE consortium. Abstract query templates that described sets of constraints in these competency questions were then developed. Queries were then instantiated by a team of curators with biomedical expertise, transformed into keyword queries (with and without query expansion), and implemented using a set of four baseline information retrieval systems. The pool of query-dataset pairs for relevance judgment was derived from the top results for each of these systems, and the resulting judgments were included in the reference standard.

## Competency questions

The starting point for construction of the queries was a set of annotated competency questions that emerged from one of the bioCADDIE consortium’s working groups. This consortium was deliberately designed to engage a broad range of community stakeholders concerned with data accessibility and re-use, and the main charge of Working Group 3 involved generating a core set of descriptive metadata for dataset indexing. This work resulted in the Data Tag Suite (DATS) model, which is described in detail elsewhere (20). During the course of generating DATS, this group reviewed use cases that had been generated by stakeholders (Supplementary Appendix S2). These use cases were drawn from multiple sources, including user-submitted use cases, and those generated during a bioCADDIE use cases workshop, held in San Diego in March of 2015. Working group members generated competency questions from these, and categorized the competency questions with respect to the metadata elements of interest (21). Two examples are provided in the element labeled ‘Competency question’ in Figure 1 (top right).

## Abstract query templates

Metadata elements were then isolated from these competency questions, providing constraints to guide the development of queries for the reference set. For example, constraints on *data type*, **biological process** and disease may be instantiated as a query for *genomic data* on **t-cell homeostasis** related to multiple sclerosis. To isolate and categorize these constraints, the set of annotated competency questions was reviewed by members of bioCADDIE Working Group 4, which was charged with providing recommendations for evaluation of the DataMed prototype. Members of this working group reviewed the competency questions to identify constraints that could inform development of queries for the reference set. Questions involving datasets containing Protected Health Information (PHI) were excluded upfront, as these did not relate to currently indexed content. Questions that required returning results other than datasets (such as software packages or drug-drug interactions) were also excluded. The most commonly occurring entity types in the remaining competency questions (*n *=* *30) were data type, disease type, biological process and organism. In addition, a number of competency questions concerned characteristics of the data sets relating to permissions and data format. A smaller number of the questions concerned surrogate indicators of dataset quality, such as funding source or number of referring publications. Based on this review, the working group recommended focusing on queries concerning the most prominently featured entity types (data type, disease type, biological process and organism). The net result was a selection of three abstract queries (Supplementary Appendix S1: annotation guidelines), each embodying sets of constraints, such as {‘data type = *x’*, ‘biological process = *y’*}.

## Instantiation of queries

These abstract queries were provided to a team of curators with biomedical expertise. For each set of constraints, the curators generated biologically relevant queries. These queries were then evaluated to ensure that relevant datasets existed within the corpus, which was accomplished by searching for datasets using the DataMed prototype, resulting a set of 137 queries. From these, 15 were selected on the basis of the constraints they embodied, with the aims of providing coverage of the most commonly occurring constraints (‘data type = *w’*,‘biological process = *x’*,‘species/organism = *y’*,‘phenotype = *z’*), and providing a range of query difficulty, which was estimated on the basis of the number of concepts and relationships included in the query. The main motivation for selecting a subset of queries concerned the resource-intensive nature of the relevance judgment process, the number of judges available with the prerequisite expertise, and the intended timeline of the challenge evaluation. A further 6 queries were selected to enable iterative development of annotation guidelines. For each of the 21 selected queries, a set of keywords was manually extracted. In the case of the 15 queries to be used for the challenge, expansions for each of these keywords were generated using DataMed’s terminology server, which provides the means to normalize and extend concepts identified in free-text queries, and is described in further detail in Ref. (4). Briefly, this was accomplished by mapping keywords to corresponding Unified Medical Language System (UMLS) concepts, and leveraging the knowledge contained in a customized subset of the UMLS to generate synonyms and other expansions. This step was motivated by the advantages shown by systems that accommodate terminological expansion in prior (document-level) biomedical information retrieval tasks (12, 13). Examples of queries are shown in Table 3, and the results of a DataMed search for one of the examples appear in Table 4.

**Table 3.** Examples of queries, showing keyword and expanded forms

Curator query	Constraint types	Keyword query	Expanded keyword query
‘Find data on the NF-KB signaling pathway in MG (Myasthenia gravis) patients’	Biological process Disease	NF KB signaling pathway Myasthenia gravis MG	NF KB signaling pathway Myasthenia gravis MG Immunoglobulin Enhancer Binding Protein Transcription Factor NF kB Ig EBP 1 Enhancer Binding Protein Immunoglobulin kappa B Enhancer Binding Protein kappaB Nuclear Factor kappa B Immunoglobulin Enhancer Binding Protein Factor Myasthenia Gravis Ocular Myasthenia Gravis Generalized Erb Goldflam disease Myasthenia gravis disorder MG Myasthenia gravis
‘Search for all data types related to gene TP53INP1 in relation to p53 activation across all databases’.	Gene Biological process	TP53INP1 p53 activation	tumor protein p53 inducible nuclear protein 1 TP53INP1 Teap FLJ22139 DKFZp434M1317 SIP TP53INP1AP53DINP1 TP53INP1B Gene p53 TP53 LFS1 tumor protein p53 Li Fraumeni syndrome

**Table 4.** Top 5 datasets retrieved in response to second query in Table 3 (DataMed, 6/10/17)

Query: keywords	Repository	Dataset title, link
‘Search for all data types related to gene TP53INP1 in relation to p53 activation across all databases’: **TP53INP1 (OR)** **p53 (OR) activation**	ArrayExpress	A large intergenic non-coding RNA induced by p53 mediates global gene repression in the p53 transcriptional response, https://www.ebi.ac.uk/arrayexpress/experiments/E-GEOD-21761/
	Uniprot:Swiss-prot	**T53I1_RAT (**Tumor protein p53-inducible nuclear protein 1 LIR Poly-Glu) http://www.uniprot.org/uniprot/Q80YE2
	BioProject	**Identification of LATS transcriptional targets in HeLa cells using whole human genome oligonucleotide micorarray**http://www.ncbi.nlm.nih.gov/bioproject/PRJNA119427
	Uniprot:Swiss-prot	T53I2_RAT (Tumor protein p53-inducible nuclear protein 2) http://www.uniprot.org/uniprot/Q8CHM3
	Sequence Read Archive (SRA)	**Genome-wide RNA-expression analysis after p53 activation in colorectal cancer cells.**https://www.ncbi.nlm.nih.gov/sra/SRX961220[accn]

## Generation of initial result pool

Four open source search engines were then used to generate a pool of datasets for relevance judgment. The search engines were selected with the aims of incorporating a range of information retrieval approaches, to mitigate the danger of the reference set being biased toward a particular established approach. The search engines used to generate the initial pool of results are described in Table 5. Of these search engines, Indri (22), Lucene (23) and Terrier (24) have been widely used in the context of TREC-style information retrieval evaluations, and are often used as baseline methods against which to compare new approaches. They cover the three main families of information retrieval methods: geometrically-motivated models (the Vector Space Model (25)), probabilistic models [e.g. Language Modeling (26)] and set-theoretic models (Boolean retrieval). Semantic Vectors (27, 28) was selected because it provides a computationally convenient way to find documents that are meaningfully related to terms in a query without their necessarily having to contain these terms explicitly. We anticipated this would further broaden the pool of results. We prepared the corpus in the required format for each of these search engines (using a locally developed python script), and applied them to index the corpus, treating the aggregated content of the ‘dataset title’, ‘description’, ‘repository name’ and ‘publication’ fields in a record as a single text document. For the 15 queries that constitute the test set for the challenge, both the keyword and the expanded queries were applied, for a total of eight runs (two per indexing system). The top 300 results from each run were retained, and these results were then merged, to eliminate duplicate datasets (i.e. identical datasets retrieved by multiple search engines–we did not address the issue of redundancy across repositories). The net result was a list containing those datasets returned within the top 300 results for each of the 15 queries. In addition, the top 100 results across systems for the 6 supplemental queries were retained.

**Table 5.** Information Retrieval systems for the initial pooling experiments

System	Description and key algorithms. Algorithms deployed to generate the pooled results for the reference standard appear in **boldface**.
Apache Lucene (https://lucene.apache.org/)	Underlies the ElasticSearch implementation used for the bioCADDIE prototype. **Vector Space model (**25**)** with capacity for Boolean logic.
Indri (http://www.lemurproject.org/indri/) (22)	**Language Models (**26**)** and Inference Networks (29).
Terrier (http://terrier.org/) (24)	**Divergence from Randomness** (30) BM25 (31).
Semantic Vectors (https://github.com/semanticvectors/semanticvectors) (27,28)	Extends Apache Lucene. Implicit query expansion via term similarity - Random Indexing (32), Latent Semantic Indexing (33) and **Reflective Random Indexing (**34**)**.

## Assessment

A team of annotators with biomedical expertise conducted relevance judgments. The initial round of evaluation involved the six additional queries, and resulted in iterative improvements to the annotation guidelines (see Supplementary Appendix S1 for the final version). In keeping with the procedures established by the TREC community for the Genomics track (12), relevance judges were asked to decide if retrieved datasets were irrelevant, partially relevant or definitely relevant to the query concerned. For a dataset to be judged definitely relevant, it should relate to all of the key concepts in a query, and capture any stated relationships between them. In contrast, partially relevant datasets might capture most but not all of the key concepts, or fail to reflect an important relationship. Relevance judges were explicitly encouraged to seek synonyms for entities in queries (such as genes and diseases), and directed toward a range of resources that could be used for this purpose (Supplementary Appendix S1). They were not provided with the automatically expanded queries. A distinguishing feature of this process is that relevance judges were also instructed to look beyond the retrieved metadata to establish relevance if necessary. Metadata provided upon deposition of publicly available datasets is often terse in nature, and may not be adequate to determine whether a dataset fits the constraints imposed by a query. So, relevance judges were encouraged to look beyond the metadata as required. For the most part, this involved exploring PubMed articles that were linked to the dataset description, where these were available. Similarly, challenge participants were instructed to look beyond the metadata provided in the corpus, in order to encourage the development of novel methodological approaches.

**Figure 2.**
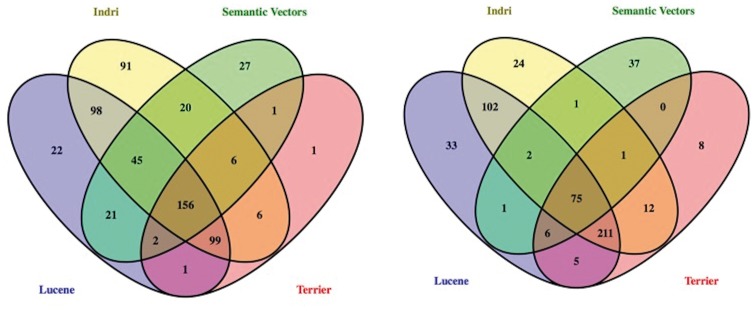
Venn diagram showing the overlap in ‘definitely relevant’ results across systems with (right) and without (left) terminology-based query expansion. Produced using Venny (36).

One hundred highly ranked datasets from across the four systems were judged for each of the six additional queries. Each of these datasets was judged by two judges with biomedical expertise–one with an undergraduate major in biology, and the other with a graduate degree in pharmacy. Agreement amongst these annotators was somewhat better than that documented in prior information retrieval evaluations (35), with an unweighted Kappa of 0.67. Any disagreements were discussed and ultimately resolved with the assistance of an adjudicator with doctoral-level training in biochemistry. The fifteen queries used for the challenge evaluation were reserved until the annotation guideline was stable. Datasets to be judged consisted of the merged top 300 results for each of the eight runs described, resulting in a pool of 18 417 relevance judgments (this is less than the total number of datasets in the top 300 runs–15 x 8 x 300 = 36 000–on account of the overlap between systems). Of these, 785 and 2787 were judged definitely and partially relevant, respectively. The Venn diagram in Figure 2 shows the overlap in ‘definitely relevant’ results produced by each search engine, with (Figure 2 right) and without (Figure 2 left) the use of terminology expansion. Though some systems overlapped more than others, documents retrieved by a single system only make up a substantive proportion of the total number of relevant documents. In the two configurations, with and without query expansion, Semantic Vectors and Indri returned the largest number of ‘definitely relevant’ documents that were not returned by any other system, respectively. This was anticipated for Semantic Vectors, on account of its application of Reflective Random Indexing (34) to identify relevant datasets that do not state query terms in their metadata explicitly. With Indri, this may be attributable to the capacity of inference networks (29) to estimate relevance on the basis of combinations of features that are otherwise viewed independently.

In addition to this initial pool, once the challenge deadline had passed, any previously un-annotated datasets present in the top 10 results for any submitted run were evaluated. A randomly selected sample of 5% of the previously un-annotated datasets ranked within the remaining top 100 results for any submitted run was also evaluated. The cumulative result of these procedures was a set of 20 184 relevance judgments for 15 queries. Of these relevance judgments 812 (∼4%), 3069 (∼15%) and 16 303 (∼81%) were judged as definitely, partially and not relevant, respectively. Of these, 27 were definitely relevant datasets and 282 were partially relevant datasets obtained from the submitted runs, and as such represent relevant datasets that were not highly ranked by the baseline methods.

## Discussion

Although this benchmark set is similar in some respects to evaluation sets for prior biomedical ad-hoc retrieval tasks, it is worth reiterating some important differences. An obvious distinction is that the ultimate targets for retrieval are datasets, not documents. Descriptive metadata for these datasets are used as surrogates for the datasets themselves, but may only approximately represent their contents. In general, with document retrieval no such separation occurs–the content of interest lies within the document to be retrieved. This is somewhat analogous to the case in which a full text manuscript is consulted to determine the relevance of an indexed abstract, but the degree of dissociation is arguably greater with datasets–the abstract is still a part of the document under consideration, but the metadata, which are the primary determinant of relevance to a search in this case, are not a part of the dataset, which may be neither index-able nor searchable with a conventional search engine. Judges were therefore often required to look beyond the retrieved metadata. Although this usually involved reviewing publications linked to a dataset, it is conceivable that exploring the dataset itself may have been of value, and the development of automated methods to interrogate datasets in this manner presents an opportunity for future research. One might imagine the acquisition of information concerning the nature of a dataset (‘gene expression data’) or some key discrepancies [‘overexpression of gene X’–tools are already available to draw such inferences, for example (37)] in this manner. One might also envision moving beyond the ‘Findability’-oriented criteria for relevance used in this challenge to include criteria that address the remaining three FAIR Guiding Principles (38)–Accessibility, Interoperability and Reusability. A reference standard that takes these factors into account would broaden the scope of system evaluation, such that both the relevance and the potential utility of datasets are taken into account. Another distinction, which is of practical importance for the development of dataset retrieval systems, involves the breadth of structured data fields available in the index, and the inconsistency with which these were populated across records. This inconsistency arises in the benchmark on account of the diverse nature of the repositories from which datasets were drawn, and cultural differences between the communities that maintain them. Within bioCADDIE, this issue is being addressed by mapping fields from different repositories to a common data model, DATS (20). However, this process was not complete at the time the reference benchmark was generated, so some of the challenge of integrating metadata fields ‘in the wild’ was retained. In addition, the entities populating these structured fields were not described with a single terminology (in contrast to the use of either ICD-9 or MeSH in prior biomedical TREC evaluations), and may have at times contained free text. The benchmark thus provides the means to assess the utility of methods for normalization and standardization of metadata [such as (7, 9)] in the context of an information retrieval task. As is described in the accompanying papers in this volume, teams participating in the 2016 bioCADDIE Dataset Retrieval Challenge used a range of approaches to address these issues, and the results from the Challenge give some insight into their respective utilities.

## Conclusion

This paper discusses the design and realization of a public reference benchmark for biomedical dataset retrieval. Although the use of such reference benchmarks for information retrieval is well established as an evaluation paradigm, the domain of biomedical dataset retrieval presents additional challenges on account of the sparse and often inconsistent nature of the metadata available in public repositories. Consequently, there is a need for the development of novel methods that can augment these metadata, either by extracting additional information from related publications, or by automated exploration of the underlying data directly. Both of these possibilities present rich and largely unexplored territory for the development of novel information retrieval approaches. Our intention is that the public release of this reference standard will facilitate the evaluation of these approaches as they emerge.

## Supplementary data

Supplementary data are available at *Database* Online. 

## Supplementary Material

Supplementary Appendix_A

Supplementary Appendix_B
