# Supplementary material for: A publicly available benchmark for biomedical dataset retrieval: the reference standard for the 2016 bioCADDIE dataset retrieval challenge
Source: Database (Oxford). 2017 Aug 18;2017:bax061. doi: 10.1093/database/bax061 (PMC5737202; doi:10.1093/database/bax061)
Supplement: Supplementary Appendix_A [file bax061_appendix_a.docx]

**Appendix A:** bioCaddie Benchmark Dataset Annotation Guidelines

June 1, 2016

This document contains the guidelines to annotate descriptions of datasets retrieved by search engines in response to queries. To annotate the dataset and indicate the relevance of the results to the question, three options have been given: **Relevant, Partially Relevant, and Not Relevant**.

Task Description

You will be presented with metadata describing datasets retrieved by a dataset search engine. These paragraphs contain the portions of text resulted from the search engine query. The queries are developed from three competency questions designed to evaluate the quality of the results. The competency questions are listed below:

1. **Search for data types x and y related to the same biological process z**
2. **Search for data types x (genome data) with biological process (mutations) y and z in species/organism a for phenotype b**
3. **Search for disease x data of all types across all databases**

| Sample Question | Target Answer | Example |
| --- | --- | --- |
| Find datasets describing the role of a gene involved in a given disease | The role of the target gene in the disease. | **Search for gene DRD4 and alcoholism disease:** Look for the role of DRD4 in alcoholism. |
| Find datasets describing the role of a gene in a specific biological process | The **effect** of the target gene on the biological process. | **Search for insulin receptor gene and tumorigenesis:** Look for what effect the insulin receptor gene has on tumorigenesis |
| Find datasets describing interactions (e.g., promote, suppress, inhibit…) between two or more genes in the function of an organ or in a disease | The type of genes’ interaction with the functionality of the organ. | Search for **HMG** and **HMGB1** and **hepatitis:** look for how do **HMG** and **HMGB1** interact in **hepatitis**. |
| Find datasets describing one or more mutations of a given gene and its biological impact | How a mutation in a gene **influences** a biological process | **Search for mutation and Ret and thyroid:** How does a mutation in Ret influence thyroid function. |

Evaluation Measures

Before you begin to judge, scan the results and look for frequently appearing terms. Look them up to see how they relate to the concepts in the question. Are they sub-topics or synonyms? There are some questions for which only a few paragraphs are relevant or partially relevant. It is tempting to show leniency when few relevant paragraphs are encountered. Resist the urge to relax criteria for relevance and try to maintain consistent evaluation standards.

Judging consistency is also affected by familiarity with the topic. As you judge results, you will become more familiar with the area of research, and may change your criteria for relevance. For unclear dataset titles and descriptions, check the publication and see if it mentions the query topic and terms. If the publication has the criteria related to the topic, mark it accordingly.

1. A dataset is **relevant** if it captures all required concepts in the question **AND** it **answers the question** or **there is a relationship between terms** or **key concepts**.
2. If **all key terms exist**, but there is no relationship between terms, mark it as **partially relevant**. In other words, a result is **partially relevant** if it contains all of the concepts but has missing elements or doesn’t answer the question. Additionally, a result is **partially relevant** if it contains the majority of concepts (2/3, or 3/4).
3. If no related concept exists, or the majority of the concepts are missing, mark the dataset as **not relevant**. E.g. if you found 1 term out of 3, the dataset will be not relevant although it mentioned one key concept. If there are 2 terms and you found 1, again mark it as not relevant because the majority in this case is finding both terms.
4. If there is a gene or protein mentioned, identify synonyms for it. For biological processes or diseases, familiarize yourself with more general concepts, as well as sub-topics. E.g. “**mad cow disease**” is formally known as **bovine spongiform encephalopathy (BSE)**. Additionally, check the content of the dataset to see if it is referring to a related disease. For example, “Creutzfeldt-Jakob Disease” (CJD) is similar to BSE and both belong to the family of diseases known as the TSEs, but they are not related to the question.
5. When annotating a question related to a disease, make sure that you understand all related concepts and synonyms that logically describe the disease. **One disease may represent multiple concepts which are not related with each other**. E.g. “**bone marrow edema**” implies **Bone Marrow Diseases** and **Edema**. The same rule implies when a similar term for a disease has been used on the dataset.
6. In another example, a question asks about **liver function**. The definition of “liver development” in the Gene Ontology states that the ***liver secretes bile***, ***synthesizes blood clotting factors*** and ***vitamin A***, and ***stores glycogen***. Those functions that are unique to the liver and are supplied as an answer are relevant. References to functions that occur in other organs in addition to the liver are partially relevant.

Examples and Reasoning for Relevance Judgments

| # | Question | Excerpt | Judgment | Reason |
| --- | --- | --- | --- | --- |
| 1 | What is the role of **PrnP** in **mad cow disease**? | Bovine Prion Protein Gene (**PRNP**) Promoter Polymorphisms Modulate PRNP Expression and May Be Responsible for Differences in **Bovine Spongiform Encephalopathy** Susceptibility | Relevant | It directly talks about the concept of the question. |
| 2 |  | **Transmissible spongiform encephalopathies (TSEs)**, or prion diseases, are mammalian neurodegenerative disorders characterized by a posttranslational conversion and brain accumulation of an insoluble, protease-resistant isoform (PrPSc) of the host-encoded cellular prion protein (PrPC) | Partially Relevant | It refers to family of disorders (**TSEs**) that includes mad cow disease. It is not clear whether findings apply to mad cow disease. (Guideline 6) |
| 3 |  | The central role of PrP in the transmissible spongiform encephalopathies (TSEs), the proximity of the gene which encodes doppel (Prnd) to the PrP gene (**Prnp**) and the structural similarity shared by PrP and doppel have led to the proposition that ataxia which develops during TSE disease could, in part, be due to doppel. | Not Relevant | It discusses the role of gene adjacent to PrnP. |
| 4 | What is the role of IDE in Alzheimer’s disease | There is an inverse correlation between in vivo insulysin activity levels and brain A peptide levels and suggest that modulation of insulysin activity may alter the risk for Alzheimer's disease. | Relevant | IDE is the gene symbol for the protein insulysin. |
| 5 |  | Taken together these results suggest that the use of insulysin to hydrolyze A peptides represents an alternative gene therapeutic approach to the treatment of Alzheimer's disease. | Relevant | IDE is the gene symbol for the protein insulysin. |
| 6 | How does APC protein affect actin assembly | We showed that APC clusters were colocalized with DLG protein at cellular protrusions of subconfluent MDCK cells. A portion of the clusters was found at the tips of microtubules extending into the cellular protrusions. In addition, actin stress fibers converged near the clusters. | Partially Relevant | APC not shown to affect actin assembly, it just co-localizes with it. |
| 7 | How does BRCA1 ubiquitinating activity contribute to cancer | Both genes contribute to homologous recombination and DNA repair, to embryonic proliferation, to transcriptional regulation and, for BRCA1, to ubiquitination. But questions regarding BRCA1 and BRCA2 biology remain, and their resolution is critical for clinical development. Why do ubiquitously expressed genes that participate in universal pathways lead, when mutant, specifically to breast and ovarian cancer? | Relevant |  |
| 8 |  | Proteasome-mediated degradation of BRCA1 protein in MCF-7 human breast cancer cells.” Is partially relevant because the proteasome is part of the ubiquitin pathway, but not limited to it. Additionally, “When monoubiquitinated, the FANCD2 protein co-localizes with the breast cancer susceptibility protein BRCA1 in DNA damage induced foci. | Partially Relevant | It links between ubiquitin on FANCD2 and BRCA1 is not specified. |
| 9 |  | Defining biochemical functions for the BRCA1 tumor suppressor protein: analysis of the BRCA1 binding protein BAP1 | Not Relevant | The biochemical functions may refer to ubiquitinating activity, but it is too vague to count as relevant |
| 10 |  | The recent identification of the ubiquitin protein ligase activity of BRCA1 implies a possible functional connection between both genes. | Not Relevant | It doesn’t mention cancer. |
| 11 | What interactions between **CFTR** and **Sec61** cause **degradation** of CFTR, leading to **cystic fibrosis** | The level of the **Sec61-CFTR** complexes are highest when **CFTR degradation** proceeds at the greatest rate which leads to **cystic fibrosis** | Relevant |  |
| 12 |  | **CFTR** expression and **ER-associated** **degradation** in yeast | Partially Relevant | **Sec61** may be part of **ER-associated** degradation apparatus |
| 13 |  | We now have identified three short oligopeptide regions in the C-terminal domain which impact **cystic** **fibrosis** **transmembrane conductance regulator (CFTR)** maturation and stability in different ways | Not Relevant | There is no mention of a possible degradation pathway |
| 14 | Search all types on **multiple sclerosis** (MS) of all types across all databases | Safety Study of Natalizumab to Treat **Multiple Sclerosis** (MS) | Relevant | Mentioned employing a technique related to MS |
| 15 |  | Impact of Vitamin A on Gene Expression, in **Multiple Sclerosis** Patient. | Relevant | Mentioned the impact of a gene expression for patient **with MS disease**. |
| 16 |  | Study of the Effect of BG00012 on MRI Lesions and Pharmacokinetics in Pediatric Subjects With **RRMS** | Relevant | **RRMS** is a similar disease in the same family. |
| 17 | Data on T-cell homeostasis related to multiple sclerosis (MS) | We investigated the regulation of the size of Interleukin-2-producing CD4+ **T-cell** (IL-2p) pool using different IL-2-reporter mice. | Not Relevant | It hasn’t mentioned **MS** and therefore, the majority of concepts do not exist. |
| 18 |  | Suppression of **T-Cell** Activation and Collagen Accumulation by an Anti-IFNAR1 mAb, Anifrolumab, in Adult Patients with **Systemic Sclerosis** | Not Relevant | Although it mentioned **Systemic Sclerosis,** it didn’t mention **MS**. |
| 19 |  | Through their functional diversification, CD4+ **T-cells** play key roles in both driving and constraining immune-mediated pathology. Polymorphisms within the locus encoding a transcription factor BACH2 are associated with diverse immune-mediated diseases including asthma, **MS**, vitiligo and type 1 diabetes. | Not Relevant | The terms exist, but MS only mentioned as an example and has no relationship |
| 20 | Data on **synaptic growth** and **remodeling** related to **glycolysis in the human brain** | PWe find that depletion of the nucleosome **remodeling** and deacetylation (NuRD) complex in the cerebellar cortex by in vivo RNAi in rats.These findings define NuRD-dependent promoter decommissioning as a developmentally regulated programming mechanism that releases the brake on presynaptic differentiation and thereby drives **synaptic** connectivity in the mammalian **brain**. | Not Relevant | The majority of the concepts and the main term (glycolysis) don’t exist, and there is no relationship between existing terms. |
| 21 |  | Aim: To gain insight into the pathways by which GABABR activation may influence long-term changes in **synaptic** *plasticity* and **neuronal growth** and morphology we investigated changes in gene expression in cultured hippocampal neurones evoked by the GABABR agonist baclofen. | Not Relevant | The majority of the concepts and the main term (glycolysis) don’t exist, and there is no relationship between existing terms. |
| 22 |  | Circular RNAs formed by the atypical head-to-tail splicing of exons, have re-emerged as a potentially interesting RNA species given recent reports of a surprising diversity and abundance of circRNA in organisms ranging from worm to **human**. Using deep RNA sequencing, we profiled different RNA species in mouse and observed that circRNAs are significantly enriched in **neural tissue**, relative to other tissues. These data indicate that **brain** circRNAs are positioned to respond to and regulate **synaptic** function. | Not Relevant | The majority of the concepts and the main term (glycolysis) don’t exist, and there is no relationship between existing terms. |

| 23 | Data on **BRCA gene mutations** and the **estrogen signaling pathway** in women with stage I **breast cancer** | Effect of Tamoxifen or an Aromatase Inhibitor on Estrogen Metabolism in Women Undergoing Treatment for Newly Diagnosed Breast Cancer. | Not Relevant | This topic doesn’t talk about BRCA gene mutation and signaling pathway. The majority of concepts don’t exist and the terms are not specific enough to be considered as synonyms. |
| --- | --- | --- | --- | --- |
| 24 |  | Identify **genes** related to the occurrence of breast events, defined as occurrence of invasive **breast cancer**, in women at high risk of developing breast cancer who have **received a Selective Estrogen Receptor Modulator**. Cases and controls were selected from the tamoxifen arm in the P-1 and from the tamixifen and raloxifene arms. | Partially Relevant | 2 out of 3 concepts exist. The dataset doesn’t talk about BRCA gene or signaling pathways, but the existing terms are potentially relevant to the question and have a relationship. |

Resources for Judging

IHoP – Information Hyperlinked over Proteins

http://www.ihop-net.org/UniPub/iHOP/

This database lists synonyms for proteins and provides excerpts from the literature, allowing you to familiarize yourself with the biology of the protein.

PubMed Books

http://www.ncbi.nlm.nih.gov/entrez/query/Books.live/Help/bookhelp.html#search

Good for general overviews of biological processes and diseases. Link takes you to instructions for searching books.

AmiGO, the Gene Ontology browser

http://www.godatabase.org/cgi-bin/amigo/go.cgi

Good for brief definitions of biological processes. No disease information. Use MeSH or PubMed books.

MeSH – Medical Subject Headings

http://www.nlm.nih.gov/mesh/MBrowser.html

For biological processes and diseases, provides synonyms or constituent processes that are part of the indicated concept.
