# Supplementary material for: A publicly available benchmark for biomedical dataset retrieval: the reference standard for the 2016 bioCADDIE dataset retrieval challenge
Source: Database (Oxford). 2017 Aug 18;2017:bax061. doi: 10.1093/database/bax061 (PMC5737202; doi:10.1093/database/bax061)
Supplement: Supplementary Appendix_B [file bax061_appendix_b.pdf]

Consolidated list of proposed bioCADDIE Use Cases

George Alter
5/25/15

Key to sources:

- **WPx = bioCADDIE White Paper**
- **UCx = breakout group at the Use Cases Workshop (**March 8, 2015**)**
- **PREx = submitted to the Use Cases Submission Form prior to the Workshop**
- **PBx = proposed by Phil Bourne (email 1/27/2015)**

A. Scientific question, premise, or problem statement.	B. Data sources and types.	C. Competency or requirements questions.
**Disease-based search across scales**		
WP1. Find all data sets from Alzheimer's patients that have RNA-seq, behavioral and imaging data available.		
UC1.<br>1. What data exists relevant to Huntington's Disease, what data types, what repositories?<br>2. What data is available that allows comparison of behavioral studies with HD and ADHD?<br>3. What datasets relating to Parkinson's, Alzheimer's, Schizophrenia and Bipolar Disorder mention HD or the HD gene?	Predict-HD<br>dbGaP	Search strategy:<br>1. Search for HD in DDI (maximum sensitivity)<br>a. will retrieve all datasets of different data types that relate to HD, and the repositories where data is located<br>2. Comparison of behavioral studies<br>a. Search DDI for: HD AND Behavioral data (or types of behavioral data)<br>b. Search DDI for: ADHD AND Behavioral data (or types of behavioral data)<br>c. DDI results for each search identifies datasets that include behavioral data for either HD OR ADHD<br>d. User then looks through datasets to see which datasets from ADHD and HD can be compared<br>3. Search involving database content<br>a. Search DDI as follows: (Parkinsons OR alzheimers OR schizophrenia OR bipolar) AND HD<br>b. Will retrieve all datasets in DDI that cover each disease/disorder as they relate to HD
UC3. Clinical research on breast cancer wants to predict cardiotoxicity of a new chemo drug<br>● what are existing drugs with a similar mechanism to the new one?<br>● what is known about 'omics correlates of cardiotoxicity for related drugs?<br>● what is the natural history of patients given similar drugs? (need EHR data, self-report/sensor data)<br>● for trial participants, want to track symptoms as they develop to ensure early detection of cardiotoxicity (MD2K sensors include lung water congestion, stroke volume, and physical activity and EMA data)	iSpy2 trial<br>ATHENA project	Metadata requirements<br>● available data elements (representation may be via ontologies and tagged using standard vocabularies)<br>● participant/patient ID scheme (to map across data sources)<br>● provenance and data lineage<br>● permissioning<br>● biosample availability and permissioning<br>● data access policies and logistics<br>● links to relevant publications, etc describing how data was accrued<br>Discovery approach<br>● Find data with patients "like these" with similar treatments/responses to treatments, similar genetically, etc.

PRE14<br>I am building a reference set of drug-drug interactions. I have downloaded existing data files from research publications and other public repositories of drug-drug interactions, and applied an algorithm to generate non-redundant set of interactions. I have prepared this data as RDF nanopublications, which already contains the DDIs, their evidence and provenance, and digital object metadata. Given the work entailed, I would like to share this data for others to find and reuse, and to cite the associated paper (under preparation).		a. automatic extraction of the structured metadata that is contained *in* my data file.<br><br>b. enabling others to find this dataset through a search / browse interface.<br><br>c. enabling others to cite my dataset and/or the associated paper.d. showing people that this work is derived from other DDI datasets, which may also be added to the DDI (and hence enabling others to avoid having to do the integration task that we have).
Pre17<br>I am studying menagement and outcomes of pregnancy and childbirth in women suffering from Factor VII congenital defficiency.		I need individual life histories with clinical parameters, and data on outcomes.<br><br>birth of a healthy baby<br>mother without clinically significant hemorrhage<br>both alive at the moment of birth and two months later without thromboembolic events
**Molecular-based search across organisms and scales**		
WP2. A user wants to get all proteomics and metabolomics data sets related to the same biological process. The data were published in different articles but refer to the same or highly related experiments. There are currently no unique identifiers for the data sets. Link to Elixir: A brief discussion of this use case occurred in the January 14, 2015 meeting related to object identifiers. This use case will be one of three driving the prototype.		
PB1.<br>User X wants to know what datasets are available that relate to gene expression analysis on mouse red blood cells.	GEO	
UC2<br>Search for Apoptosis across organisms and scale	Genbank, PDB, Uniprot, GO, PRIDE, ProteomeXchange, Metabolights, Reactome, PathwayPORTALS, TCGA, COSMIC, David, COPaKB, Human Proteome Atlas	Each resource needs to provide metadata that describes content:<br>● Organism<br>● biological process<br>● scale, including modality<br>● estimate of reliability of the annotations<br><br>The DDI is expected to run queries across multiple repositories.
PRE18<br>I am a new researcher in the field of the unfolded protein response (UPR) to cell stress. I wish to identify previously uncharacterized		Literature searches are useful for identifying already published pathways regulating UPR, but less so for uncharacterized ones. The lack of organization of transcriptomics-scale datasets in the field of cell signaling makes searching of individual supplemental data files or GEO datasets very time consuming. There is no way to

signaling molecules and transcription factors that regulate the expression of genes involved in the UPR.		search across datasets in an unbiased manner. Annotation of datasets is patchy and inconsistent, and data are presented in a variety of unpredictable formats.<br><br>It will point me to a resource where transcriptomic datasets are curated and organized to facilitate searching and comparison across multiple datasets in an unbiased manner.
**Molecular data/phenotype associations**		
WP3. A user wants to know what datasets are available that have genome data about IDH1 and IDH2 in humans or other species for a particular phenotype of interest (e.g., glioma).	dbGAP	● A search on the DDI would return datasets that have glioma cases with information on IDH1 and IDH2.<br>● The GA4GH currently is sending a query for genome hosting institutions to respond (Yes/No) via its Beacon initiative, which does not yet involve phenotypes.<br>● The query could be significantly extended to include counts and certain phenotypes.
PB2<br>User X is returned multiple datasets that meet the search criteria, all appear useful - which to try and use?		There is no way to determine this currently, but one way in which the DDI could help is when an entry is accessed a web service call is sent to the source and access statistics returned. Access statistics are an example of metadata which is dynamic. Rather than being collected once when the dataset is uploaded; it is collected whenever the dataset is reviewed from the DDI.
UC5<br>What is the best (according to “my” criteria) or most relevant data I can use that can inform which of my 1000 variants is causal for Autism?	1000 genomes<br>dbGAP<br>ClinVar<br>FMRI	1. (Relevance) Find exome data of neurotypical and autism patients.<br>a. Identify fMRI data of patients in previous dataset<br>2. (Relations- enhancement) Find existing datasets where 1000 genomes and fMRI a data have been linked/integrated via patient identifier<br>3. (Relations- subsetting) Find dataset that was subsetted based upon vaccination history<br>4. (Trust) Filter for datasets by NIH researchers with more than 100 papers in autism research, and were peer reviewed
PRE1<br>Describe the impact of drug and alcohol use on the developing brain and identify phenotypes that can be used by physicians to preempt drug and alcohol abuse.	combination of clinical, neuropsychological and multimodal brain imaging technologies to identify phenotypes that are predictive of alcohol and drug use disorders	
PRE2<br>I am studying the effects of genetic variation on brain structure and function in healthy adults, or in people diagnosed with various psychiatric disorders.	dbGAP, NIF, SchizConnect	Ideally I need neuroimaging data (structure or resting state fMRI) across the lifespan, with deep phenotyping (cognitive measures, clinical symptom measures, basic demographics and personality measures, family histories) and genome-wide scan (GWS) data.<br><br>Identifying which variables are the same and which are different across studies (how did they screen for "healthy controls"? Which question is which on which clinical measure? Is this measure of IQ the same as some other from another study?).
PRE9<br>I am seeking patterns in the genome and transcriptome that are indicative of response to drugs		● A biomarker that has a high accuracy in predicting drug response<br>● Documentation of variables and experimental conditions

**Behavioral and environmental data**		
WP4. A user wants to know "*What is the effect of stress on health? Could different components (family, work, neighborhood) have stronger associations with health?"*	ICPSR	● Using the DDI, which indexes data elements and instruments that measure stress from different life experiences, the user searches for "stress at work."<br>● The search brings up variables used in surveys as well as NIH Common Data Elements.<br>● Using facets to narrow the search, she finds survey questions that measure the concept that she has in mind.
UC4.<br>What are the effects of behavior change interventions on patients with BMI>35 and Diabetes?	ClinicalTrials.gov	• Searching by BMI<br>• Screening facets<br>– Age, gender, ethnicity<br>– Drugs e.g. metformin<br>– Behavior change
PRE3<br>I am studying how the effect of educational attainment on adult mortality in the United States has changed historically. I need measures of educational attainment that are harmonized over time as well as mortality follow up. I also need to identify individuals' birth cohorts.	ICPSR	1) datasets that contain measures of educational attainment linked to mortality follow up;<br>2) possible harmonized datasets with mortality information.
PRE8<br>I am trying to assess tobacco product use behaviors across a wide population of users and products as well as risk perceptions, changes in behavior over time, and changes in health outcomes over time.	ICPSR	To do this, I need survey instruments and data as well as data on biological markers in those subjects. Ideally, these would include longitudinal data.

[PRE-WORKSHOP USE CASE SUBMISSIONS]

[USE CASES FROM WHITE PAPER]

[USE CASES FROM PHIL]

[USE CASES FROM BREAKOUT GROUPS AT THE MARCH WORKSHOP]

[LINK TO USE CASE WORKSHOP NOTES]

[LINK TO WHITE PAPER]

From White Paper

i. Disease-based search across scales

Find all data sets from Alzheimer's patients that have RNA-seq, behavioral and imaging data available.

ii. Molecular-based search across organisms and scales

A user wants to get all proteomics and metabolomics data sets related to the same biological process. The data were published in different articles but refer to the same or highly related experiments. There are currently no unique identifiers for the data sets. Link to Elixir: A brief discussion of this use case occurred in the January 14, 2015 meeting related to object identifiers. This use case will be one of three driving the prototype.

iii. Molecular data/phenotype associations

A user wants to know what datasets are available that have genome data about IDH1 and IDH2 in humans or other species for a particular phenotype of interest (e.g., glioma). Here a Google search would return articles or informational web sites that refer to data sets, some of them available in dbGAP. A search on the DDI would return datasets that have glioma cases with information on IDH1 and IDH2. Link to BD2K: The GA4GH currently is sending a query for genome hosting institutions to respond (Yes/No) via its Beacon initiative, which does not yet involve phenotypes. The query could be significantly extended to include counts and certain phenotypes. This use case will be one of three driving the prototype.

iv. Behavioral and environmental data

A user wants to know "*What is the effect of stress on health? Could different components (family, work, neighborhood) have stronger associations with health?"* A Google search results in thousands of articles, but the researcher will need to read each article to determine if it is based on relevant data. Using the DDI, which indexes data elements and instruments that measure stress from different life experiences, the user searches for "stress at work." The search brings up variables used in surveys as well as NIH Common Data Elements. Using facets to narrow the search, she finds survey questions that measure the concept that she has in mind. This use case will be one of three driving the prototype.

v. Precision Medicine

We will not address precision medicine use cases to comply with NIH's directive.

FROM WORKSHOP

Four use cases that were written for the White paper were discussed at the Use Case Workshop

1. Disease-based search across scales: Find all data sets from Alzheimer's patients that have RNA-seq, behavioral and imaging data available.
2. Molecular-based search across organisms and scales: A user wants to get all proteomics and metabolomics data sets related to the same biological process. The data were published in different articles but refer to the same or highly related experiments. There are currently no unique identifiers for the data sets.
3. Molecular data/phenotype associations: A user wants to know what datasets are available that have genome data about IDH1 and IDH2 in humans or other species for a particular phenotype of interest (e.g., glioma). Here a Google search would return articles or informational web sites that refer to data sets, some of them available in dbGAP. A search on the DDI would return datasets that have glioma cases with information on IDH1 and IDH2. Link to BD2K: The GA4GH currently is sending a query for genome hosting institutions to respond (Yes/No) via its Beacon initiative, which does not yet involve phenotypes. The query could be significantly extended to include counts and certain phenotypes.
4. Behavioral and environmental data: A user wants to know "What is the effect of stress on health? Could different components (family, work, and neighborhood) have stronger associations with health?" A Google search results in thousands of articles, but the researcher will need to read each article to determine if it is based on relevant data. Using the DDI, which indexes data elements and instruments that measure stress from different life experiences, the user searches for "stress at work." The search brings up variables used in surveys as well as NIH Common Data Elements. Using facets to narrow the search, she finds survey questions that measure the concept that she has in mind.

FROM PHIL BOURNE

From Phil Bourne email dated: 1/27/2015 Agreement

User X wants to know what datasets are available that relate to gene expression analysis on mouse red blood cells. How would they address that now? Either they would search the literature for associated papers from which data sets may or may not be available or they would go to known data sources e.g. GEO. Neither would be satisfactory. Thus the long term goal would be to have a single index to look up where appropriate data are available. Within the scope of a three year project the goal would be have enough information automatically collected from a collection of websites/resources to begin to address such a question.

User X is returned multiple datasets that meet the search criteria, all appear useful - which to try and use? There is no way to determine this currently, but one way in which the DDI could help is when an entry is accessed a web service call is sent to the source and access statistics returned. Access statistics are an example of metadata which is dynamic. Rather than being collected once when the dataset is uploaded; it is collected whenever the dataset is reviewed from the DDI. These features should be addressed within a 3-year prototype.

FROM PRE-USE CASE WORKSHOP SUBMISSIONS

bioCADDIE Use Case Workshop – March 8, 2015

Ref	Actor (Who are you?)	Your discipline or field	Describe the use case.	Describe a successful outcome	Assuming the data you want exist, what are the main barriers to finding those data?	How can the Data Discovery Index (DDI) make it easier to identify relevant data?	First Name	Family/Last Name
1	Researcher	Human Brain Imaging	I am studying the drug and alcohol naive adolescent brain using a combination of clinical, neuropsychological and multimodal brain imaging technologies to identify phenotypes that are predictive of alcohol and drug use disorders.	Describe the impact of drug and alcohol use on the developing brain and identify phenotypes that can be used by physicians to preempt drug and alcohol abuse.	It is difficult to find data with sufficient metadata and quality control measures to know if it is actually usable. Further it is difficult to know what all the specific measures mean that need to be processed...Most data is poorly curated. Also, some datasets are not readily available because investigators will not make it available.	I think my providing standard descriptions of data in my domain and potentially providing tools that researchers can use to appropriately annotate/organize their data.	Nolan	Nichols
2	Researcher	psychology, psychiatry, neuroscience	I am studying the effects of genetic variation on brain structure and function in healthy adults, or in people diagnosed with various psychiatric disorders. Ideally I need neuroimaging data (structure or resting state fMRI) across the lifespan, with deep phenotyping (cognitive measures, clinical symptom measures, basic demographics and personality measures, family histories) and genome-wide scan (GWS) data.	A successful outcome would be the ability to identify genetic profiles which covary with greater or less expression of various brain structure or function patterns in the individual, with the ability to run models of the mediating effects of environmental and other individual variables.	Knowing where to search for it--no one makes their data available through a single portal (dbGAP is a beginning, as is NIF and SchizConnect).<br><br>Identifying which variables are the same and which are different across studies (how did they screen for "healthy controls"? Which question is which on which clinical measure? Is this measure of IQ the same as some other from another study?).	work with other international efforts to standardize semantics, collate synonyms, extract assertions/interpretations, and push for easier methods for everyone to share their data.	Jessica	Turner
3	Researcher	Demography	I am studying how the effect of educational attainment on adult mortality in the United States has changed historically. I need measures of educational attainment that are harmonized over time as well as mortality follow up. I also need to identify individuals' birth cohorts.	I am not sure what is meant by a successful outcome. If my guess is correct, a successful outcome would be a DDI that allows me to identify: 1) datasets that contain measures of educational attainment linked to mortality follow up; 2) possible harmonized datasets with mortality information.	I am guessing that the main barriers are: 1) inconsistent coding of educational attainment; 2) coding educational attainment in a way that prevents an assessment of the effects of the "tails" of the educational distribution (less than high school and college degree or more).	One stop shopping to identify the possible "population" of datasets.	Mark	Hayward

4	Researcher	Bioinformatics	We want to provide a way for scientists at Cornell to query broader and updated phenotypic categories for generalized enrichment analysis on 'omics data. Existing gene set enrichment annotation tools rely on curated annotations based on custom categorization, and can't keep up with ever increasing 'omics literature. Increasing GEO and ArrayExpress profiles / signatures that correlate with phenotype or outcome, are not easily queryable from existing tools. To enable scalable, relevant enrichment analysis incorporating new study profiles, DDI could help standardize ontologies for self- and crowd-sourced annotation of these study data.	A successful implementation of "live" enrichment analysis would provide a sort of "phenotype search" based on measured profiles (variants/genes, proteins, metabolites, etc) similar to the annotative searches that BLAST and similar tools provide for sequence data.	GEO and ArrayExpress provide various levels of 'omics data linked to publications - each has an API which allows profile retrieval. However, identification of relevant sets is keyword oriented and rarely yields useful / complete coverage of studies.	To enable the use case described above, the DDI could define a queryable representation for "study profiles" (phenotype associated sets), where both the phenotypic and the profile variables (gene, protein, metabolite, etc) are organized within a useful subset of standard ontologies (MIAME/MGED, ICD, HPO, etc). This would enable "semantic" retrieval of related profiles of interest. The DDI team(s) could focus on curating the data model (upload format) and the phenotype and profile taxonomies, and rely on crowd-sourcing for specific profile/set curation by study researchers and the 'omics community.	Hanif	Khalak
5	Researcher	Oncology	Goal is to enable access to the molecular biology of carcinogenic pathways for clinicians who are interested in resolving clinical questions with regards to a specific patient. Plan is to link a virtual networking environment, Cancer Commons (Cancercommons.org) to biomedical data and clinical trials that is relevant to the cancer (subtype) under discussion, and - where possible - the genetic mutations known to be relevant for patient cohorts.	We are interested in enabling easy access to knowledge in all relevant research (gene/protein/pathway) and clinical (trials, patient-reported outcomes, where possible) data and, where necessary, to the corresponding literature, in an open, social platform. The goal is to allow access to knowledge in an online environment that enables discussions between oncologists, radiologists, pathologists and medical researchers, which is also accessible to the patients.	1) Knowing which data to query2) Identifying relevant knowledge components in the most pertinent databases. 3) Understanding how to access them4) Integrating them into one easy to use platform.	Collaboration with various (NIH and external) databases would be essential - it would be great if the DDI could help create a central place to query and link to appropriate datasets.	Anita	de Waard
6	Researcher	data visualization	I am studying the various constraints of genotypes and phenotypes. I need to know what people look for when they search for genotypes and phenotypes	A list of various constraints and root index for phenotypes and genotypes	No direct data source available.	Guide to discover how my data discovery can improve regarding genotypes and phenotypes	vidya	Narayana

7	Researcher	Health Informatics	Use case 1. Pharmacovigilance with electronic health records (EHRs)<br>To actively monitor side effects after drugs are released into the market, EHR data are used as a data source for pharmacovigilance research. Many side effects are rare, so multiple EHRs may be needed to achieve sufficient power. For each EHR, it is important to know clinical context, data quality, prevalence of medication use and side effects, etc.<br><br>Use case 2. Pharmacovigilance by combining EHRs with knowledge bases<br>When identifying possible adverse drug reactions from EHR systems, improved performance may be achieved by adding knowledge about their possible mechanisms. Drug-related knowledge is distributed in heterogeneous information sources, such as SemMedDB, DrugBank, and PharmGKB. Therefore, the ability to link EHR results with such knowledge bases would be useful.<br><br>Use case 3. Treatment pathways<br>To investigate patients' treatment status over the course of a patient's disease, real-life treatment options that were experienced by patients can be analyzed by using EHR data. Clinical data at each phase of treatment over the pathway are needed, such as disease progression, treatment change and discontinuation, outcome of the patients, and hospital setting.		Find and access appropriate EHR/CDW data for research.	Indexing EHR/CDW characteristics to assist researchers to find relevant data source.	Ning	Shang
8	Researcher	epidemiology research	I am trying to assess tobacco product use behaviors across a wide population of users and products as well as risk perceptions, changes in behavior over time, and changes in health outcomes over time. To do this, I need survey instruments and data as well as data on biological markers in those subjects. Ideally, these would include longitudinal data.	A successful outcome would be a validated description of tobacco product use behavior in a certain population, accompanied with risk perceptions and biological data in order to assess relationships between those variables.	The data are often in different databases and locations, which requires multiple searches across multiple platforms.	Having a standardized search engine with standard search terms would be very useful in identifying the databases and publications that are relevant to the research project.	Heather	Kimmel
9	Researcher	Cancer	I am seeking patterns in the genome and transcriptome that are indicative of response to drugs	A biomarker that has a high accuracy in predicting drug response	Data requires data sharing agreements etc. Also quite difficult to find the data if the variables and experimental conditions are poorly documented by the submitting researchers	Not sure	John	Watkins

10	Researcher	Informatics, Primary Care Medicine	I am with the MD2K BD2K Center. We are collaborating with the Translational Genomics BD2K Center, an autism patient group, and We are Curious, Inc a platform for citizen science and self-discovery, to combine genomic and mobile health data. Autism patients will be collecting 23&Me, microbiome, and data from commercial sensors (e.g., Apple Watch). The goal is to export that data from the Curious platform to our respective BD2K compute platforms. Data will be exported via the Global Alliance and Open mHealth APIs, which are being aligned and semantically integrated.	A researcher should be able to explore the data sources that the autism cohort draws from (e.g., which genomic test, which sensors, what data was collected), query for # of autism patients with specific genomic, microbiome, and sensor data profiles, and export the patient-level data from those patients to a "big data" compute platform. There may be publications coming out of this later, but linking this data to publications is a very secondary concern right now.	In this use case, we know where the data is and that it exists. The generalized problem is to be able to discover finer-grained details about the dataset, for cohort discovery and to see whether a request for the patient-level data is needed.	- the DDI should be compatible with domain-specific APIs, e.g., Global Alliance for genomics and Open mhealth for mobile health<br>- there should be a computable declaration of privacy preferences (we will be working with Sage on this)<br>- would be great if the DDI and associated projects have a search interface and visualization that data sources can feed into<br>- a mechanism is needed to uniquely identify data sets and individual patients (the autism community has a GUID system)	Ida	Sim
11	Researcher	Population health	I want to be able to see snippets of code in SAS or SPSS that will help me extract or combine specific variables from one large (or multiple) dataset(s).	A DDI record for a dataset would point me to useful snippets of code that I could run on that dataset (or datasets) to analyze a specific set of variables.	Simply searching for the data in general. Knowing that it is out there.	Describe datasets in a way that makes them easily retrievable (e.g. subject specific). Point me to related datasets of the same nature when I find a dataset that I like or that I can use.		
12	Researcher	Public health	I want to gain access to specific datasets, but don't know what is required of me to do this. For example, what kind of license do I need? Is the dataset free to use? Do I need to fill out an application? Will I require IRB approval? What are the data use agreements for specific datasets that need to be addressed?	Datasets in the index provide detailed information about how to access a dataset directly -- preferably narrative and clearly explained. Also, would be excellent to limit a search to datasets that are freely available, and would require minimum effort to access. Similarly, finding and limiting a search to datasets that have different requirements (e.g. limiting to IRB only, specific data use agreement data, etc.) would be helpful.	Understanding the access restrictions -- as described above.	See successful outcome above.		
13	Researcher	biomedical informatics	I am studying the genetic basis for aging and aging-related processes. I have mined a variety of datasets and identified new candidate genes that have good supporting evidence [1]. I would like to share my findings so that others can discover and experimentally validate them.<br><br>[1] http://www.biomedcentral.com/1471-2105/16/40	Links to experimental validation of our computational predictions. Knowing how many people looked at the page and downloaded the data. Being able to query for the dataset. Being able to retrieve results contained in the dataset.	a common repository for research data. easy to use interfaces to construct useful metadata. a way to integrate our findings into other, larger, and more heavily curated sources (e.g. wormbase gene ontology annotations).	1. data repository<br>2. metadata annotation<br>3. search and filter facility<br>4. submission to boutique databases<br>5. linking experimental validation of computational findings	Michel	Dumontier
14	Researcher	biomedical informatics	I am building a reference set of drug-drug interactions. I have downloaded existing data files from research publications and other public repositories of drug-drug interactions, and applied an algorithm to generate non-redundant set of interactions. I have prepared this data as RDF nanopublications, which already contains the DDIs, their evidence and provenance, and digital object metadata. Given the work entailed, I would like to share this data for others to	Users of the DDI are able to find my dataset and reuse it in downstream analyses. They will be provided a citation for the associated publication so that they can include it in their methods. I will obtain data citation statistics that I can report as part of describing the impact of my research.	a common data repository. adequate metadata to search with.	a. automatic extraction of the structured metadata that is contained *in* my data file.b. enabling others to find this dataset through a search / browse interface. c. enabling others to cite my dataset and/or the associated paper.d. showing people that this work is derived from other DDI datasets, which may also be added to the DDI (and hence enabling others to avoid having to do the integration task that we have).	Michel	Dumontier

			find and reuse, and to cite the associated paper (under preparation).					
15	Researcher	Medicine	Combining individual patient data from multiple clinical trials, from academia and industry.	Access to the analyzable data set, the protocol, and other metadata, ability to combine the data in a meaningful way, and ability to reproduce the original findings and to combine the data for new findings. Please see the IOM report Responsible Strategies for Sharing Clinical Trial Data at http://www.iom.edu/Reports/2015/Sharing-Clinical-Trial-Data.aspx for the full use case.	See the IOM report Responsible Strategies for Sharing Clinical Trial Data at http://www.iom.edu/Reports/2015/Sharing-Clinical-Trial-Data.aspx for discussion of the barriers.	There needs to be a unique ID for each clinical trial (probably the NCT number), and a sub-id for each sharable data set from the trial (the full analyzable data set, the CSRs, the analyzable data set underlying publications, etc as detailed in the IOM report). The DDI needs to work with what will likely be a global distributed federated system controlling access to clinical trial data.	Ida	Sim
16	Researcher	Medical Informatics	I am building causal discovery software and APIs that allows a user or developer to perform causal discovery and analyses over large datasets. The algorithms in the software identify causal relationships between variables in data.	Researchers utilize causal discovery software to discover unforeseen connections among -omic components of the cell. Developers incorporate our APIs into other software.	Data needs to be annotated well such that researchers can identify datasets relevant for causal analysis in their domain. Linking datasets together (additional metadata, variables or patients) us to discover and verify causal relationships not previously identified.	The DDI will provide users of Causal Discovery algorithms with a ready to use repository or links to repositories containing data for analysis. The DDI will provide users with the necessary annotations for indexed datasets to identify additional data that will augment their existing datasets. These annotations should provide variable descriptions, anonymous patient linkage information. Datasets and annotations should be in standard formats.	Jeremy	Espino
17	Clinician	Clinical pharmacology	I am studying menagement and outcomes of pregnancy and childbirth in women suffering from Factor VII congenital defficiency. I need individual life histories with clinical parameters, and data on outcomes.	birth of a healthy baby<br>mother without clinically significant heamorrhagy<br>both alive at the moment of birth and two months later without thromboembolic events	If I need to know details I need that have not been described in the documents I can reach, I have to find and contact people who have dealt with similar cases. It is totaly up to them if they want to share data with me or not, especially when studies are hidden behind the paywalls.	Make connections between keywords and individual (raw) patient data and researchers.<br><br>Facilitate search of raw data.	Mersiha	Mahmić-Kaknjo
18	Researcher	Regulation of gene expression	I am a new researcher in the field of the unfolded protein response (UPR) to cell stress. I wish to identify previously uncharacterized signaling molecules and transcription factors that regulate the expression of genes involved in the UPR. I will use this information as the basis for additional experiments, the results of which will be included as preliminary data in an R01 grant application.	A successful outcome would be to identify 2-3 pathways that regulate at least two of the genes involved in the UPR. Identifying crosstalk between these pathways would be an additional	Literature searches are useful for identifying already published pathways regulating UPR, but less so for uncharacterized ones. The lack of organization of transcriptomics-scale datasets in the field of cell signaling makes searching of individual supplemental data files or GEO datasets very time consuming. There is no way to search across datasets in an unbiased manner. Annotation of datasets is patchy and inconsistent, and data are presented in a variety of unpredictable formats.	It will point me to a resource where transcriptomic datasets are curated and organized to facilitate searching and comparison across multiple datasets in an unbiased manner.	Neil	McKenna

19	Researcher	clinical trials and public health	By using the methodology of observatory (natural experiment), and with the team, I am studying the impact of transition of transparency of clinical trial data on clinical trials, analysing opportunities and barriers.<br>The IMPACT Observatory monitors changes regarding data sharing of a) relevant policies and regulations, b) the culture of researchers and c) data repositories, will inform and indicate trends.<br>The summary of the study can be found on:<br>http://ottawagroup.ohri.ca/disclosure.html	The observatory becomes a hub of clinical trial data transparency and related changes of clinical trials; it becomes a tool in creation or revision of data sharing policies and changes of the clinical trial enterprise. It is funded by funders and other users<br>Clinical trials are changing towards broader data sharing re-analysis (re-use). Methodology of data sharing and re-use is more sophisticated, standards and guidelines are developed. The network of collaborators (information providers) expands and the information input increases<br><br>Identified as a useful tool the IMPACT Observatory is supported by all involved constituencies from researchers to regulators. They use information hosted by the Observatory in their ongoing trial data sharing initiatives and provide information on changes of their relevant policies as they arise.	Policies on transparency are not easily found- grey literature, internal documents; they might change without a warning.. Information about interactions and mutual influences of players are not easily found<br><br>Non collaborabortiive players, financial constraints	Complement DDI, by developing standards and portal to find/ identify data for re-analysis. Provide more substance a contribute/ develop methodologies and standards of what is needed to have data available for re-use/ re-analysis.<br>All researchers, starting with the NIH funded, present up front their data sharing plan, and data are assigned a DOI.and can be found via DDI	Karmela	Krleza-Jeric

Breakout Group 1.

Use Cases Workshop (March 8, 2015)

_Breakout

Participants:

- David Eichmann
- Kevin Read
- Hua Xu
- Richard Gonzalez
- Alyson Yao
- Jennie Larkin

A trio of use cases relating to Huntington's Disease. These are designed to target at minimum a known multimodal resource (Predict-HD) and it's appearance in resources such as dbGaP (11 results for HD). They also increase in likely implementation challenges as they go up. The NINDS Common Data Elements also has a CDE for HD. Other HD potential resource sites include:

- http://chdifoundation.org
- http://www.euro-hd.net/html/network?eurohdsid=e2fc2ed4ca74a0ab7324ec6481a14307

Use case questions:

1. What data exists relevant to Huntington's Disease, what data types, what repositories?
    a. Search strategy:
        i. Search for HD in DDI (maximum sensitivity)
            1. will retrieve all datasets of different data types that relate to HD, and the repositories where data is located

2. What data is available that allows comparison of behavioral studies with HD and ADHD?
    a. Search strategy:
        i. Search 1: Search DDI for: HD AND Behavioral data (or types of behavioral data)

ii. Search 2: Search DDI for: ADHD AND Behavioral data (or types of behavioral data)
1. DDI results for each search identifies datasets that include behavioral data for either HD OR ADHD
a. User then looks through datasets to see which datasets from ADHD and HD can be compared

3. What datasets relating to Parkinson's, Alzheimer's, Schizophrenia and Bipolar Disorder mention HD or the HD gene?
   a. Search strategy:
      i. Search DDI as follows: (Parkinsons OR alzheimers OR schizophrenia OR bipolar) AND HD
         1. Will retrieve all datasets in DDI that cover each disease/disorder as they relate to HD

Vocabularies/ontologies required to describe the above concepts:

Concept 1: Disease/Disorder/Topic

Example Vocabs: MeSH, ICD-9

- MeSH: "Huntington Disease"
- ICD-9: (333.4) Huntington's chorea

Concept 2: Data type

Example vocabs: Biosharing, others??

Example of what this would look like:

Behavioral data (major concept)

Apathy evaluation scale (subconcept)

Cambridge Brain Repair Centre (BRC) HD Sleep Questionnaire (subconcept)

Hospital Anxiety and Depression Scale (subconcept)

Breakout Group 2.

Use Cases Workshop (March 8, 2015) Participants:

- Helen Berman
- Anders Garlid
- Peipei Ping
- Howard Choi
- Christina Kendziorski

Search of biological processes across organisms and scale

Using Apoptosis as example biological process

1. Identified key resources that would provide information

Genbank, PDB, Uniprot, GO, PRIDE, ProteomeXchange, Metabolights, Reactome, PathwayPORTALS, TCGA, COSMIC, David, COPaKB, Human Proteome Atlas

2. Also discussed lab and boutique resources, “small r” repositories

3. Each resource needs to provide metadata that describes content

Organism, biological process, scale, estimate of reliability of the annotations

4. Recommendation: bioCADDIE should ask repositories to fill in metadata items that are required. Small resources would have to conform to the format that bioCADDIE provides in order to be included

Breakout Group 3.

Use Cases Workshop (March 8, 2015)

Participants:

- Ida Sim
- Heidi Sofia
- Anita deWard
- Julia Puzak
- Dawei Lin
- Lucila Ohno-Machado
- Ian Fore
- Karmela Krleza-Jeric

Summary (from Ida):

Assuming target user is a clinical researcher in breast cancer, at the hypothesis generating stage of the research process. bioCADDIE website should support DDI of an initial seed query, then support iterative data-driven browsing and query refinement, ie bioCADDIE needs to support the *process* of data discovery where the user can tune sensitivity/specificity to their specific needs. The initial bioCADDIE webpage could allow queries in major classes of data (e.g,. genomics, imaging, clinical (ie data from clinical care), person-generated (ie sensors, apps), public health, and environmental (maybe also regulatory)). Metadata that will be needed for data sets across all these domains include:

- available data elements (representation may be via ontologies and tagged using standard vocabularies)
- participant/patient ID scheme (to map across data sources)
- provenance and data lineage
- permissioning

- biosample availability and permissioning
- data access policies and logistics
- links to relevant publications, etc describing how data was accrued

Specific use case: iSpy2 trial (UCSF, part of Haussler's CBDTG Center) wants to predict and track cardiotoxicity of new chemo drugs.

- what are existing drugs with a similar mechanism to the new one?
- what is known about 'omics correlates of cardiotoxicity for related drugs?
- what is the natural history of patients given similar drugs? (need EHR data, self-report/sensor data)
- for trial participants, want to track symptoms as they develop to ensure early detection of cardiotoxicity (MD2K sensors include lung water congestion, stroke volume, and physical activity and EMA data)

The UC-wide ATHENA project has clinical, genomic, and imaging data of about 80,000 women (?), and has just gotten a PCORI grant that involves substantial patient participatory research.

Explicit resources should be apportioned to defining a clear set of queries on reference data sets to develop competency questions for evaluating retrieval performance. User-centered evaluation, where the iteration of queries is user-driven, can complement system evaluation.

Julia's Notes:

Ida- specific use case: use the need for eye spy 2 researchers for adaptive studies for early therapeutics for breast cancer. All patients have clinical data, genome/exome data, testing new therapeutics from companies, tracking cardiotoxicity. Want to have real time sensing and symptomatic data- coordinating with clinical research data. What are other therapeutics that have similar mechanisms of action? Could be considered in a learning healthcare context.

Anita- specific use case: expose what is known on the molecular side about a particular cancer. Getting output with access to case reports and clustering of outcomes after a particular treatment that perhaps correlate with something on the molecular side. Traverse those two types of data. Cancer commons, DARPA. Virtual tumor board.

Any recommender system- focused on a medical use case. Have patients “like these” with similar treatments/responses to treatments, similar genetically, etc. Pivot all of these knowledge sources.

Primary user- clinical researcher, oncologist- explore given a history and a patient, what do we know about this that may offer a good direction to look at? There are data known about these aspects. Look for certain types of data exist- connect with data in other studies.

Will only know if the variable is collected or not, not what the data is. Hypothesis generated clinical research. I want these variables as I have a hypothesis about how these data fit together. Need to be able to find metadata/permissioning, size of dataset, demographics of patients, biosamples/permissioning, imaging/modality of imaging AND if other datasets that are available, does the repository participate in a data sharing- rules and scale to sharing/access. Is blue button available, what kind of participant ID scheme they are using, provenance issue- is this data interoperable, what identifier system is being used and is it the same across datasets? Identification of a dataset vs. the components. In order to answer the clinical question, need all of the components. Each component is indexed/packaged and there is a link to other components. Need to identify policies for sharing datasets- community governance. People put data into university repository- problem of how to link publication with data- publishers are creating linkages to datasets (link-data infrastructure through NSF pilot), authors needs to expose the links through stable identifiers.

What do users want from publications? Molecular side. Combine with the beacon strategy- do the patients have that variant. Want to determine characteristics associated with patients- what kind of modality.

Finding datasets- retrieval considering sensitivity and specificity. Do we want to have a search result to be very sensitive, specific, or general? Want to be very sensitive initially and then results returned will be progressively more specific.

DDI is the beacon answer- rank on how many they have, rank with yes permissions, rank with yes phenotypes, etc.

Workflow: Paper on IDH1 and IDH2- mutation in IDH1 is relevant to cancer X, go to TCGA DCC- mutation has been validated in these particular genomes (somatic mutation is relevant to a particular cancer)- beacon will give you germline instances/heredity- DDI will help you find which data are the most relevant/indicate the value of datasets before requesting data.

Each database that returns from the beacon broadcasts basic metadata. Metadata about the data you've touched on. Choose strategically which you should request. What is the feature set within bioCADDIE that lets you do it like PubMed and combine searches A and C and exclude search B.

Data on women who do not have cancer in research- will the data be comparable- some may be applicable. Biorepository index- do you have the control patients.

Ranking, faceted search issue. Need a thinly populated knowledge base or to be able to filter a thickly populated knowledge base.

Understanding where beacon is transmitting to. Biohaystack- addressed concept of websites that are transmitting what they are about, started with what you're interested in and looking at things that you had identifiers that were relevant to your query and pulled them in. Repositories are exposing themselves with agreed upon metadata.

RDF wrappers, beacon- responsibility of DDI and have the repositories adopt this.

Use case: put effort to bridge gaps and demonstrate that data need to be indexed because we can do this. More people will see the value. Attracts more users. Leverage investment made in repositories- ICPCR- take advantage of the work that has already been done. Focus is on what other components are needed (data types at particular modalities)- are they available now- linked to formulate different types of queries- easy test case, but need to fix the disconnects- demonstrate what can be connected to answer a particular question.

Clinical research data that is not necessary in a large repository.

Website- iterative browser that helps people to ask the right questions, because of the data they get to the correct data/question. What is the intention of user to come to website? What type of data do I want to find- imaging, genomic, etc.? What are the metadata available about these? Querying a large number of databases- when do you know when you've got the right results/ideal outcome. Need to handcraft particular outcomes- experts know which datasets should be found, whereas the novice doesn't know what they're looking for.

Phase before the prototype with a user interface. Dynamic real time interactive browser. Yes, I can ask and get a result on my initial question. What is the next iteration? Big loops and small loops- need to define which loops bioCADDIE can do and do well. Processing may need to be done on the data before you do the next iteration, but not necessarily. Query, exploration process- short term.

Search breast cancer- click boxes of imaging, genomics, and carcinogenic pathways, etc.

Which studies have we tested this drug in? Need to index studies by drug. What was the investigative agent and the role of the agent? Connecting drug ontologies, ontologies specifying the roles, and molecular mechanism

One end to end mock up- reverse engineer one use case- identify a breast cancer that we can find an answer to- make sure all of the components are there. Is it possible for the DDI to test the system?

Very possible to translate this particular use case to other diseases besides breast cancer.

Reference dataset that we have for testing- need to make sure that extrapolates to unseen datasets.

Breakout Group 4.

Use Cases Workshop (March 8, 2015)

Participants

- George Alter
- Ron Margolis
- Peipei Ping fellows

- Clinical investigation: search by patient data
- Obesity (e.g based on BMI)
- User: clinician with patients seeking information about studies

User requirements

- The clinician wants to know whether there are studies that include obesity patients (BMI >35) that either have interventions or completed studies
- Goal: to find studies to which an obese patient could enroll to lose weight or data from completed studies where interventions have been proven effective and the results are now in the clinic

Outcomes

- Searching from BMI reveals ~3000 hits
- Screening to reduce to manageable #
  - Facets base e.g. age, gender, ethnicity

  - Re-screening e.g. metformin? Behavior change?

- What is the size of the result that allows the user to reasonably understand outcomes?
- What level of granularity is needed to achieve focus?

Breakout Group 5.

Use Cases Workshop (March 8, 2015)

Participants:

- Jeremy Espino
- Susanna Sansone
- Carol Bean
- Melissa Haendel

Two biology problems:

1. What is the best (according to "my" criteria) or most relevant data I can use that can inform which of my 1000 variants is causal for Autism?
2. Find datasets related to obesity (defined by bmi) and diabetes

Finding Data

User considerations for deciding which data sets to gather/use:

- Relevance to their topic or interest
  - Need contextual relevance for how the data was created in order to determine relevance, need this for data to become a first class findable object. More than column and row definitions.
  - what conclusions were drawn from the data? What was the hypothesis of the experiment?
- Trust or quality of the source (both the dataset and the database hosting it)
  - Which databases are the data from? Are the data curated, what is the licence?
  - Which community standards (minimal reporting requirements, terminologies etc) if any, were used to curate and enrich the description of the dataset?
  - Need to understand the origin of the source (see pathguide figure below, for example).
  - Community usage and evaluation, reputation, metrics (quality indicators) of the database and datasets (and their descriptions)
  - Has the dataset been peer reviewed by a journal or other means?

- Attribution/credit to any prior manipulations or creation
    - licensing
    - attributions for any modifications, scrubbing, annotation
    - Who funded the data creation? its modification?
- Relations amongst datasets
    - Derivation - what was done to it? a link is good but not enough info to make a judgement. We don't expect full provenance of every element, e.g. a full diff between every integration or modification (this would be a full workflow implementation), but conversely a simple "derives_from" link isn't quite enough to make decisions regarding relevance, trust, attribution, or other facets.
    - Enhancement - What was done is something that one might not therefore have to do later - versioning is critical to KNOW whether or not something needs enhancement/fixing/scrubbing etc
    - Subsetting - Ability to identify derived portions of data or modified data uniquely (a handle for such things)

For variant analysis, what possible datasets:

1000 genomes

dbGAP

ClinVar

FMRI

Competency questions:

1. (Relevance) Find exome data of neurotypical and autism patients.
    a. Identify fMRI data of patients in previous dataset
2. (Relations- enhancement) Find existing datasets where 1000 genomes and fMRI a data have been linked/integrated via patient identifier
3. (Relations- subsetting) Find dataset that was subsetted based upon vaccination history
4. (Trust) Filter for datasets by NIH researchers with more than 100 papers in autism research, and were peer reviewed

5. (Attribution- curation) Find data sets that were curated according to a standard X or by Researcher Y, or by project B.
6. (Attribution - licensing) Only show datasets that I am able to redistribute under an academic license for free.

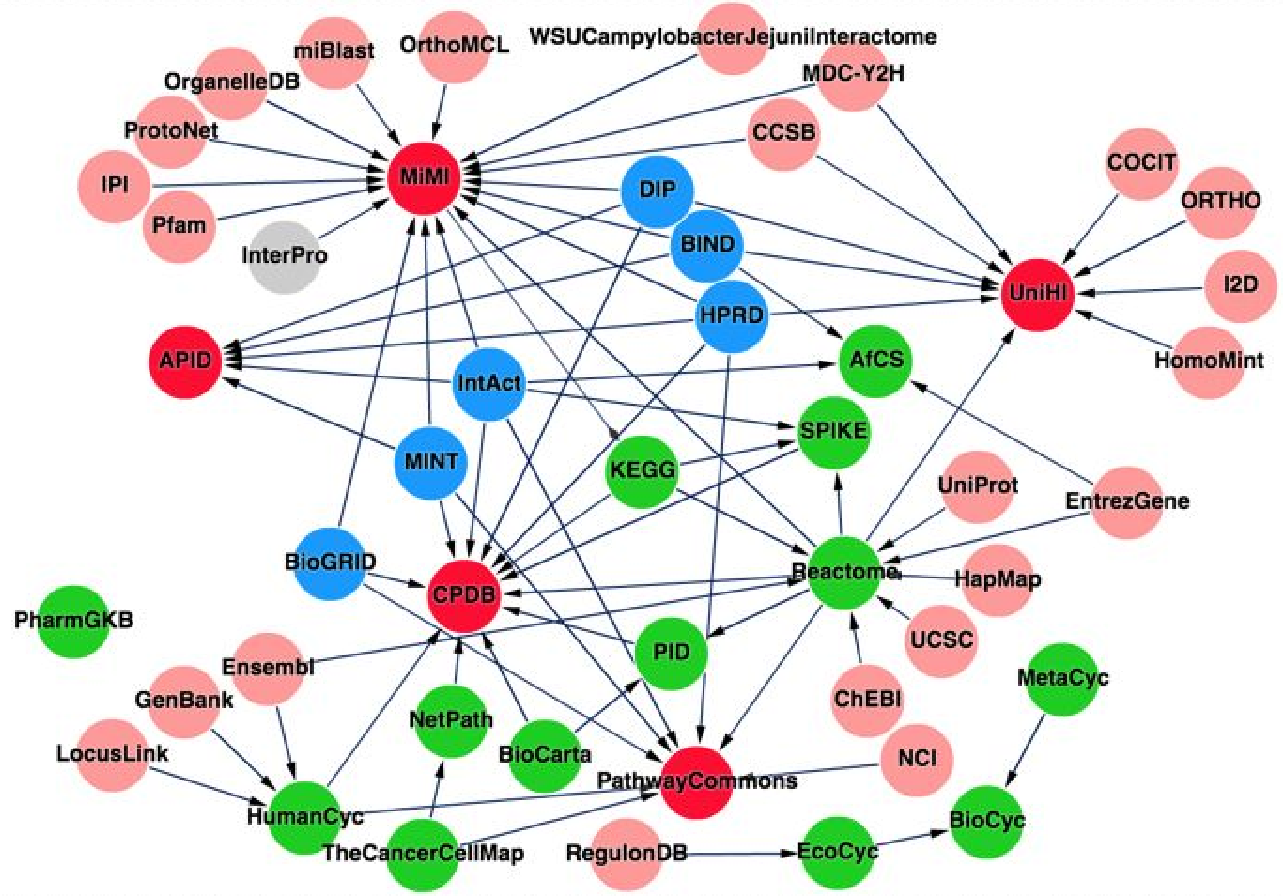

Sample use case structure:

A. Scientific question, premise, or problem statement. Here one must pose some kind of scientific inquiry. This initial scoping can be broad or narrow. A narrow example could be "I would like to understand the correlation of Lithium in the groundwater with psychiatric outcomes of patients with bipolar disorder family history." A more broad example might be "I would like to understand the effects of diet on diseases of the liver." General goals or problem statements can be listed here, such as: "drug treatment of psychiatric illness is confounded by environmental variables such as groundwater chemicals and diet, these need to be taken into account during treatment decisions." Use cases can be biological in nature, social, or technical.

B. Data sources and types. Include the following details for each data source that will be leveraged to address the above problem statement. Reference use of any existing data standards/formats or vocabularies/ontologies.

1. **Source**: Person or organization that "owns" the data (include link and/or citation)

a. **Description**: Describe the nature of the study/project that generated the data (e.g., Patient study data).

b. **Type**: Describe what the data represents (e.g., Dietary preferences).

c. **Format/data details**: Describe the format of the data and use of any standardized identifiers, vocabularies, or other data standards (e.g., Diet items are recorded using a self-defined controlled vocabulary using RedCap, the list is here: link, the amounts are recorded as times per week).

d. **Sample data**: Here you would include for each source/type listed above, a row or example of the data. Highlight components that conform to standards.

e. **Gap(s)**: List potential data gaps or limitations to the data set. If the missing data is essential, provide a comment about how this limitation will be addressed.

Note that for sources with multiple Types, each Type should be listed with its own format/data details, sample data and gaps unless this information is consistent for all Types.

C. Competency or requirements questions. Provide questions that will be enabled by integration and alignment of the above data. Questions should range from very simple to more complex. They will be used to design the data integration structure and determine downstream functionality. This list can be short at first, but lists of questions over a hundred are not uncommon when designing a semantic structure to be utilized within a search system. These questions are analyzed for semantic components and data availability/integrity as well as to test system function. Include the source/person that provided the question, as you will return later to see if the results are as they expected. Examples:

1. Search for patients who drink soft drinks.
2. Search for patients with liver inclusion phenotypes and who drink more than 4 drinks/day.
3. Search for diseases that are correlated with mutations in nuclear hormone receptor genes and patients with significant exposure to environmental estrogens.
